# Public Health Threat Assessment of Vehicular Load Index-Induced Urban Air Pollution Indices Near Traffic Intersections In Central India

**DOI:** 10.7759/cureus.11142

**Published:** 2020-10-24

**Authors:** Raghvendra Gumashta, Aanchal Bijlwan

**Affiliations:** 1 Community Medicine, People's College of Medical Sciences and Research Centre, Bhopal, IND

**Keywords:** air pollution, assessment, particulate matter, public health, traffic

## Abstract

Objectives: To assess traffic vehicular load, levels of various air pollutants, their correlation at selected traffic intersections of Bhopal city and to suggest suitable public health measures.

Methods: A transverse study was conducted by convenience sampling with equated distribution among vehicular load-based large (Group1:G1: 10 TI), medium (Group2:G2: 5 TI), and small (Group3:G3: 5 TI) traffic-intersections (TI) through a systematic stratified random selection of study sites to assess traffic vehicle load index (VLI).

Results: VLI,G1 (cumulative mean: 16.31; day-time (DT): 19.03, DT range 11.68-51.49; night-time (NT): 13.59, NT range 11.7-18.0), VLI,G2 (cumulative mean: 0.965; DT:0.971, DT range 08.56-11.67; NT: 0.960, NT range 07.54-11.39), and VLI,G3 (cumulative mean: 06.17; DT:06.08, DT range 04.12-06.86; NT: 06.27, NT range 03.74-07.53). There is a significant intergroup difference of the mean (G1 vs G2: p=0.03); (G1 vs G3: p=0.002); (G2 vs G3: p=0.003). The range of VLI is found to be wide within G1 (DT; 11.68-51.49; NT 11.7-18.00) as compared to narrow range in G2 (DT; 8.56-11.67; NT7.54-11.39) and G3 (DT; 4.12-6.86; NT 3.74-7.53).

Conclusion: High air pollution noted at TIs and associated exposure to unprotected commuters pose public-health risks. It has long-term health consequences requiring focused multidisciplinary preventive interventions.

## Introduction

Air pollution has been posing long-term, medium-term, and short-term challenges since long to the public health authorities thereby adversely affecting air quality indices [1]. PM10 standards for the ecologically sensitive area as per National Ambient Air Quality Standards is 60 µg/m^3^ for an annual time-weighted mean of a minimum 104 measurements in a year at a particular site taken twice a week, 24 hourly at uniform intervals. PM10 standards for 24 hours time-weighted mean is 100 µg/m^3^. There is a felt need to revise PM10 standards to ensure avoidance of air pollution through preventive interventions at multiple levels with a special focus on maternal and child health [2].

Many studies have conducted air pollution indices in an indoor environment, but the studies related to the outdoor environment are much needed [3-8]. In 2015, Government of India, together with IIT Kanpur launched the National Air Quality Index. In 2019, India launched "The National Clean Air Programme" with a tentative national target of 20-30% reduction in PM2.5 and PM10 concentrations by 2024, considering 2017 as the base year for comparison. It will be rolled out in 102 cities that are considered to have air quality worse than the National Ambient Air Quality Standards. The present study will identify the traffic load wise pollution indices and their interrelationship. Hence, this study shall enrich the understanding of the vehicular pollution load in Central India as per the important pollution indicators. 

We hypothesize that the analysis of various pollutants viz. (PM 2.5, TVOC, CO_2_, HCHO, PM1.0, PM 5.0, PM 10, and submicron particles) influence outdoor air quality. These pollutants are a threat for enhanced health risks especially for the vulnerable population including the elderly, children, and pregnant women. Therefore, the purpose of this study is to assess traffic vehicular load, levels of various air pollutants, and their correlation at selected TIs of Bhopal city and to suggest suitable public health measures. This study will also provide recent most air pollution scenario in moderately populated Bhopal city for possible timely preventive interventions.

## Materials and methods

A transversal study was conducted at a Tertiary Health Care Centre, Bhopal, India from June to September, 2019 to assess traffic vehicular load, levels of various air pollutants and their correlation at selected TIs of Bhopal city, and to suggest suitable public health measures. 

Sample collection

The systematic stratified random selection of the TIs was conducted by convenience sampling with equated distribution among load-based large (Group1-G1), medium (Group2-G2), and small (Group3-G3) TIs (total 20). Vehicle load index (VLI) was assessed using "vehicular indices calculation matrix datasets." The vehicle load was determined herein by the exclusive VLI developed in this study assuming the load of each two-wheeler, three-wheeler, cars, medium vehicle, and the heavy vehicle being 1.0, 1.5, 2.0, 2.0, and 6.0, respectively. The number of vehicles passing through each studied TIs was assessed lane wise as a mean of three readings at day-time (DT) and night-time (NT) for three days inclusive of last day being the reading date for all the air pollutants using calibrated Ambee Air Quality Monitor TM. The total number of TIs (n=20) included in the study were distributed in high traffic areas (G1: n=10), medium traffic area (G2: n=05), and low traffic area (G3: n=05). These included signaled (S) TIs (G1:07; G2:05; G3:01), semi-signaled (SS) TIs (G1:01; G3:01), non-signaled (NS) TIs (G1:02; G3:03S) (Tables 1 and 2).

**Table 1:** VLI in the DT and NT at TIs of groups G1, G2, G3 in Bhopal city during June-September, 2019 (n=20) #(S)=signaled TI: (NS)=non-signaled TI: (SS)= semi-signaled TI. TI: traffic intersection, VLI: vehicle load index, DT: daytime, NT: nighttime.

S.No.	TI sites (S/SS/NS) #	VLI		TI site mean	TI group mean		
		DT	NT		DT	NT	Overall mean
1	Arera colony (S)	51.49	18.00	34.74	19.03	13.59	16.31
2	Chetak bridge (S)	19.76	17.23	18.49			
3	New market (S)	18.83	13.97	16.4			
4	Shivaji nagar (S)	16.94	13.34	15.14			
5	Anand nagar (NS)	15.53	12.67	14.1			
6	Old city (NS)	15.01	12.48	13.7			
7	Ambedkar Square (S)	14.52	12.3	13.4			
8	Lalghati (S)	14.24	12.28	13.26			
9	Piplani (SS)	12.34	11.94	12.14			
10	Polytech (S)	11.68	11.7	11.69			
G2: Total 05 TIs: Medium traffic areas							
11	Bus st. (S)	11.67	11.39	11.53	09.71	09.60	09.65
12	Karond (S)	09.98	10.1	10.04			
13	Chowk (S)	09.29	09.69	09.54			
14	Habibganj (S)	09.07	09.31	08.155			
15	Railway station (S)	08.56	07.54	08.05			
G3: Total 05 TIs: low traffic areas							
16	Gandhi nagar (S)	06.86	07.53	07.19	06.08	06.27	06.17
17	Mandi (SS)	06.83	06.88	06.85			
18	Narela (NS)	06.55	06.83	06.69			
19	Ayodhya (NS)	06.07	06.38	06.22			
20	Kasturba (NS)	04.12	03.74	03.93			

**Table 2:** Group-wise comparison of VLI with gaseous particles and particulate matter (n=20) TI: traffic intersections, VLI: vehicle load index, TVOC: vehicle load index, DT: daytime, NT: nighttime.

Groups	VLI		TVOC		CO_2_		HCHO		PM10		Submicron particles (>0.25, >0.3, >0.5, >10) present at (no. of TI/total TI)
	DT:NT ratio	Group mean	DT:NT ratio	Group mean	DT:NT ratio	Group mean	DT:NT ratio	Group mean	DT:NT ratio	Group mean	
G1	1.40	16.31	2.45	0.73	1.30	1170.75	2.69	1.91	2.50	320.03	47/50
G2	1.01	9.65	0.52	1.40	1.19	921.90	0.75	0.14	0.09	43.44	24/25
G3	0.96	6.17	3.66	0.16	1.18	830.30	3.00	0.02	0.53	145.97	23/25

Measurement of pollution parameters

Various pollutants PM2.5, TVOC, CO_2_, HCHO, PM1.0, PM5.0, PM10, particle size >0.3 µm, >1.0 µm, >2.5 µm, >5.0 µm, >10 µm, temperature, dew point, humidity, ultraviolet, and visibility were recorded in DT and NT at all selected TIs using calibrated Ambee Air Quality Monitor^TM^ (Ambee Pvt., Ltd., Mumbai, IND). Hence, the study variables included particulate matter: PM (PM 2.5, PM10, PM1, PM5); gaseous pollutants (CO_2_, HCHO, TVOC); submicron particles (>0.25 µm, >0.3 µm, >0.5 µm, >10 µm). In addition to the above, the meteorological conditions prevailing on the days of data collection included temperature, humidity, dew, wind speed, air pressure, ultraviolet radiation, and visibility. The inclusion criteria for the study were randomly selected ten large, five medium, and five small traffic transactions. The convenient sampling was done twice a day for three consecutive days at each of the randomly selected sites. The list of these categories was prepared based on the pilot study undertaken for the overall load of traffic transactions at ten intersections of Bhopal distributed in all of its geographical zones. Those not listed in the randomly selected list of traffic transactions were excluded from the study. The portable digital device used for assessing various pollution parameters in the study was Ambee Air Quality Monitor^TM^.

Study sites

Twenty TIs were chosen in Bhopal city as per inclusion and exclusion criteria. Based on the traffic load, these TIs were divided into large, medium, and small. Various atmospheric pollutants studied included PM 2.5, PM 10, HCHO, CO_2_, etc., were monitored using Ambee Air Quality Monitor^TM^. Vehicle at the intersections was further divided into two-wheeler, three-wheeler, cars, heavy, and medium vehicles. Traffic load at each lane of each of the TIs was calculated for three consecutive days with the last day being data collection day for meteorological and air pollution parameters as well. The VLI was assessed as described above. The study was carried out at DT from peak traffic hours of 10 AM to 12 PM and at night from 6 PM to 8 PM. Random quick interviews were also conducted among pollution under control (PUC) check service provider, car service agencies, roadside tyre puncture repair shops, and traffic constables in each of these categories (G1, G2, G3).

Statistical analysis

All data entered were analyzed using SPSS (Statistical Package for Social Science) Version 20 (IBM Corp, Armonk, USA).

## Results

Cumulative values of VLI

VLI,G1 (cumulative mean: 16.31; DT: 19.03, DT range 11.68-51.49; NT: 13.59, NT range 11.7-18.0), VLI,G2 (cumulative mean: 0.965; DT: 0.971, DT range 08.56-11.67; NT: 0.960, NT range 07.54-11.39), and VLI,G3 (cumulative mean: 06.17; DT: 06.08, DT range 04.12-06.86; NT: 06.27, NT range 03.74-07.53). The TI site mean for G1 ranged 11.69-34.74, whereas the same for G2 and G3 were 8.05-11.53 and 03.93-07.19, respectively (Table 1). There is significant intergroup difference of the mean (G1 vs G2: p=0.03); (G1 vs G3: p=0.002); (G2 vs G3: p=0.003) (Table 3) The range of VLI is found to be wide within G1 (DT: 11.68-51.49; NT: 11.7-18.00) as compared to narrow range in G2 (DT: 8.56-11.67; NT: 7.54-11.39) and G3 (DT: 4.12-6.86; NT: 3.74-7.53).

**Table 3:** Intragroup and intergroup Spearman’s correlation coefficient for the observed noticeable values of importance (PM10, PM2.5, PM1, TVOC, CO2, HCHO, and VLI) (-) = not significant; (+) = significant. TVOC: total volatile organic compounds, PM: particulate matter, VLI: vehicle load index.

S. No.	Description	Group	Spearman correlation (rs)	p-value (two-tailed)	Significance
1	VLI vs PM2.5	G1	-0.46	0.17	-
		G2	-0.6	0.28	-
		G3	0.21	0.73	-
		G1+G2+G3	-0.43	0.05	+
2	VLI vs PM10	G1	0.12	0.72	-
		G2	-0.8	0.1	-
		G3	0.79	0.11	-
		G1+G2+G3	-0.14	0.55	-
3	PM2.5 vs PM10	G1	0.28	0.42	-
		G2	0.9	0.03	+
		G3	0.4	0.5	-
		G1+G2+G3	0.49	0.02	+
4	PM1.0 vs PM10	G1	0.87	0.00	+
		G2	0.8	0.1	-
		G3	0.5	0.39	-
		G1+G2+G3	0.71	0.00	+
5	PM1.0 vs PM2.5	G1	0.53	0.1	-
		G2	0.9	0.03	+
		G3	0.6	0.28	-
		G1+G2+G3	0.59	0.01	+
6	PM10 vs TVOC	G1	-0.04	0.9	-
		G2	0.1	0.87	-
		G3	-0.35	0.55	-
		G1+G2+G3	-0.25	0.27	-
7	PM10 vs CO_2_	G1	0.21	0.55	-
		G2	0.1	0.87	-
		G3	0.1	0.87	-
		G1+G2+G3	0.33	0.15	-
8	PM10 vs HCHO	G1	0.5	0.13	-
		G2	-0.66	0.21	-
		G3	-0.22	0.71	-
		G1+G2+G3	0.05	0.82	-

Measuring air pollution indices

The air pollution indices measured at DT and NT included gaseous particles (TVOC, CO_2_, HCHO) particulate matter (PM1.0, PM2.5, PM5, PM10) and submicron particles (>0.3 μm, >1.0 μm, >2.5 μm, >5.0 μm, >10 μm) at all 10, 05, and 05 TIs of G1, G2, and G3, respectively. The group-wise range of values for air pollution indices are indicated for DT and NT pollution level (Tables 4 and 5).

**Table 4:** Air pollution indices at DT (10 AM to 12 PM) during June-September, 2019 in Bhopal city (n=20) TI: traffic intersection, TVOC: total volatile organic compounds, VLI: vehicle load index.

S No.	TIs	VLI	PM 2.5	TVOC	CO_2_	HCHO	PM 1.0	PM 5.0	PM 10	>0.3 µm	>1.0 µm	>2.5 µm	>5.0 µm	>10 µm
1	Arera colony	51.49	26.1	1.02	1121	16.7	31	38.2	2720	320	112	30	10	3
2	Chetak bridge	19.76	7.1	1.06	4043	0.15	3.77	8.15	8.3	710	198	19	6	2
3	New market	18.83	1.35	0.17	1182	9.81	17.4	18.2	1617	189	56	18	7	5
4	Shivaji nagar	16.94	21.12	1.61	627	0.23	0.97	1.25	2.09	24	9	0	0	0
5	Anand nagar	15.53	41.6	0.09	996	0.01	18.8	45.1	45	2720	540	175	56	28
6	Old city	15.01	0.48	0.09	737	0.01	0.32	0.41	0.42	115	1	0	0	0
7	Ambedkar Square	14.52	43	1.61	1346	0.23	37.5	80.3	87.9	6568	854	244	72	36
8	Lalghati	14.24	63.3	0.16	568	0.02	12.5	37	31.7	4170	262	59	16	4
9	Piplani	12.34	36.2	0.5	2047	0.07	16	36	35.3	2765	5512	144	51	19
10	Polytech	11.68	30.8	4.08	578	0.58	10.8	25.6	28.9	2081	322	109	28	8
11	Bus stand	11.67	40.8	0.42	767	0.06	37.4	53.6	54.3	6023	505	59	15	0
12	Karond	9.98	26.1	0.42	2555	0.06	12.5	37.6	44.5	2207	497	209	61	38
13	Chowk	9.29	21.3	1.34	714	0.1	0.09	1.33	0.66	54.2	15	10	0	1
14	Habibganj	9.07	0.11	2.53	946	0.36	0.14	0.38	0.4	38	4	2	0	0
15	Railway station	8.56	27.4	0.22	648	0.03	16	21.9	21.5	2698	151	10	0	0
16	Gandhi nagar	6.86	37.4	0.09	578	0.01	20.3	44.4	46.1	2970	599	111	17	1
17	Mandi	6.83	22.5	0.3	1225	0.04	14.6	46.4	50.3	3026	727	337	126	54
18	Narela	6.55	32.3	0.09	727	0.01	154	35.9	37.7	2584	440	94	9	6
19	Ayodhya	6.07	5.51	0.56	797	0.08	4.45	11	11.8	758	112	43	3	0
20	Kasturba	4.12	30.2	0.1	878	0.01	0.06	17.8	32.3	140	327	109	16	0

**Table 5:** Air pollution indices at NT (6 PM to 8 PM) during June-September, 2019 in Bhopal city (n=20) TI: traffic intersection, TVOC: total volatile organic compounds, VLI: vehicle load index.

S. No.	TIs	VLI	PM 2.5	TVOC	CO_2_	HCHO	PM 1.0	PM 5.0	PM 10	>0.3 µm	>1.0 µm	>2.5 µm	>5.0 µm	>10 µm
1	Chetak bridge	18	8.75	0.86	1651	0.12	6.57	10.5	10.7	1350	120	26	4	0
2	Anand nagar	17.23	32.4	0.35	977	0.05	17	40.4	42.9	2790	520	133	39	15
3	Shivaji nagar	13.97	12.3	1.35	1176	0.19	9.81	17.4	18.2	1617	189	56	10	2
4	New market	13.34	1.35	o.19	1176	9.81	17.4	18.2	1617	189	56	18	7	0
5	Piplani	12.67	72.9	0.79	1186	0.11	33.3	74.2	87.9	6637	940	374	141	53
6	Lalghati	12.48	10.4	0.09	648	0.01	6.15	12.7	13.3	1080	165	37	11	9
7	Ambedkar square	12.3	4.5	0.07	578	0.01	9.11	13.87	14.02	187	32	1	0	0
8	Old city	12.28	37.2	0.04	865	0.02	3.71	5.1	0.33	108	1	0	0	0
9	Polytech	11.94	2.96	0.09	568	0.01	0.74	1.24	1.25	200	12	0	0	0
10	Arera colony	11.7	23.1	0.43	1345	0.06	10.5	17.9	18.5	1712	181	24	3	2
11	Karond	11.39	39	0.07	638	0.01	8.84	20.2	43.7	1385	294	78	21	54
12	Bus stand	10.1	27.8	6.11	875	0.54	22.5	28.9	43	1345	321	35	8	4
13	Habibganj	9.69	14.3	0.42	1440	0.03	10.6	19.2	20.3	1990	212	58	10	2
14	Chowk	9.31	19.38	2.41	738	0.21	0.11	1.03	1.01	57	11	14	10	0
15	Mandi	7.54	28.9	0.27	1022	0.02	11.4	50.2	1151	642	449	126.4	42	12
16	Gandhi nagar	7.53	28.3	0.05	607	0.01	30.2	247	58.7	1990	628	79	11	0
17	Railway station	6.88	22.3	0.1	392	0.01	17	20	205	2440	134	11	1	0
18	Ayodhya	6.83	12.1	0.1	732	0.01	7.9	8.9	7.2	1101	38	0	0	0
19	Narela	6.38	29.9	0.01	805	0.01	11.4	28.9	40.4	977	374	71	11	2
20	Kasturba	3.74	27.8	0.08	932	0.01	0.03	0.16	24.2	116	242	98	10	0

Cumulative range values of gaseous particles, particulate matter, and submicron particles

The cumulative range values of DT and NT were also assessed (Table 5). The noticeably high-value levels among groups (G1, G2, G3) were found for the following parameters (as per observed TI/total TI: G1: TVOC - 7/10; CO_2 _- 8/10; HCHO - 6/10; PM10 - 2/10, and submicron particles at all sites for all sizes ranging >0.3 μm to >10 μm except at 3/50 readings; G2: TVOC - 3/5; CO_2 _- 4/5; HCHO - 3/5; PM10- 1/5, and submicron particles at all sites for all sizes ranging >0.3 μm to >10 μm except at 1/25 readings; G3: CO_2 _- 4/5; PM10 - 1/5 and submicron particles at all sites for all sizes ranging >0.3 μm to >10 μm except at 2/25 readings). It is further observed from group-wise data that CO_2_ level is found increased in all the groups almost at all sites except 4/20, whereas TVOC and HCHO are not found increased in G3. TVOC and HCHO are found generally raised in G1 and G2 with cumulative non-observance in 5/15 sites and 6/15 sites, respectively. The TIs with the market area have essentially shown PM10 to be high irrespective of group categorization of the concerned site (Table 6).

**Table 6:** Cumulative values of DT and NT gaseous particles, particulate matter, and submicron particles against observed vehicular load as assessed by VLI in Bhopal city during June-September, 2019 (n=20) TI: traffic intersection, TVOC: total volatile organic compounds, VLI: vehicle load index.

TIs	VLI	PM 2.5	TVOC	CO_2_	HCHO	PM 1.0		PM 5.0		PM 10		PM2.5/PM10			>0.3 μm		>1.0 μm		>2.5 μm		>5.0 μm		>10 μm
Arera colony	31.5	24.6	0.72	1233	8.38	24.2		28.05		1369.25		0.87			6		146.5		27		6.5		2.5
Chetak bridge	18.85	7.9	0.96	2847	0.13	5.17		9.325		9.5		0.84			1030		159		22.5		5		1
New market	16.08	1.35	0.17	1179	9.81	17.4		18.2		1617		0.07			189		56		18		7		2.5
Shivaji nagar	15.4	16.7	1.48	901.5	0.21	5.39		9.325		10.14		1.79			820.5		99		28		5		1
Anand nagar	14.1	37	0.22	986.5	0.03	17.9		42.75		43.95		0.86			2755		530		154		47.5		21.5
Old city	13.6	18.6	0.06	801	0.01	2.015		2.755		0.37		6.75			111.5		1		0		0		0
Ambedkar Square	13.4	23.7	0.84	962	0.12	23.305		47.085		50.96		0.5			3377.5		443		122.5		36		18
Lalghati	13.3	36.8	0.12	608	0.01	9.325		24.85		22.50		1.48			2625		213.5		48		13.5		6.5
Piplani	12.5	54.5	0.64	1616.5	0.09	24.65		55.1		61.60		0.98			4701		3226		259		96		36
Polytech	11.8	21.3	2.08	573	0.295	5.77		13.42		15.07		1.58			1140.5		167		54.5		14		4
Group mean: G1: [(TVOC:0.73); (CO_2_: 1170.75); (HCHO:1.91); (PM10:320.03)]																							
Bus stand	10.8	34.3	3.26	821	0.30		29.95		41.25		48.65		0.83	3684		413		47		11.5		2	
Karond	10.6	32.5	0.24	1596.5	0.03		10.67		28.9		44.10		1.12	1796		395.5		143.5		41		46	
Chowk	18.3	20.3	1.87	726	0.15		0.1		1.18		0.83		17.2	55.6		13		12		5		0.5	
Habibganj	18.32	7.2	1.47	946	0.19		5.37		9.79		10.35		0.73	1014		108		30		5		1	
Railway street	7.7	24.8	0.16	520	0.02		16.5		20.95		113.25		1.18	2569		142.5		10.5		0.5		0	
Group mean: G2: [(TVOC: 1.40); (CO_2_: 921.90); (HCHO: 0.14); (PM10:43.44)]																							
Gandhi nagar	7.1	32.8	0.07	592.5	0.01		25.25		145.7		52.40		0.22	2480		613.5		95		14		0.5	
Mandi	7.1	25.7	0.28	1123.5	0.03		13		48.3		600.65		0.53	1834		588		231.7		84		33	
Narela	6.4	31.1	0.05	766	0.01		82.7		32.4		39.05		1.04	1780.5		407		82.5		10		4	
Ayodhya	6.4	8.8	0.33	764.5	0.04		6.175		9.95		9.50		1.13	929.5		75		21.5		1.5		0	
Kasturba	3.9	29	0.09	905	0.01		0.045		8.98		28.25		3.22	128		284.5		103.5		13		0	
Group mean: G3: [(TVOC: 0.16); (CO_2_: 830.30); (HCHO: 0.02); (PM10:145.97*)]																							
Cumulative mean of all TIs: (G1+G2+G3): [(TVOC:0.75); (CO_2_: 1023.42); (HCHO: 0.99); (PM10: 207.37)]																							

TVOC (cumulative 0.75; range 0.05-3.26; G1: 0.73; G2: 1.40; G3: 0.16); CO_2_ (cumulative 1023.42; range 520-2847; G1: 1170.75; G2: 921.90; G3: 830.30); HCHO (cumulative 0.99; range 0.01-9.81; G1: 1.91; G2: 0.14; G3: 0.02); PM10 (cumulative 207.37; range 0.37-1617; G1: 320.03; G2: 43.44; G3: 145.97; Table 6). However, PM2.5 was acceptable in most groups. Particle size >0.3 μm, >1.0 μm, >2.5 μm, >5.0 μm, and >10 μm were high. The 95% confidence interval of individual sample mean G1 (12.1016-20.5184), G2 (7.8580-11.442), G3 (4.5519-7.7880) is also depictive of calculated significant p-value (0.001365). The 95% confidence interval assuming equal variance for G1 (13.17-19.44), G2 (4.20-15.09), G3 (0.72-11.61) has F-statistics of value 9.9695 (Table 7). The significance of inter group difference of the mean is observed as: (G1 vs G2: p=0.03); (G1 vs G3: p=0.002); (G2 vs G3: p=0.003) (Table 3).

**Table 7:** ANOVA of VLI for comparison within and among the groups (n=20) ANOVA: analysis of variance, CI: confidence interval, VLI: vehicle load index, SE: standard error.

TI groups	Sample size (n=20)	Mean + SD	SE	95% CI of the individual sample mean	95% CI assuming equal variance	F-statistics	p-value
G1	10	16.31 + 5.88	02.11	12.10 - 20.51	13.17 - 19.44	9.9695	0.001365
G2	05	09.65 + 1.44	00.64	07.85 - 11.44	4.20 - 15.09		
G3	05	06.17 + 1.30	00.58	04.55 - 7.78	0.72 - 11.61		

Intragroup correlation

The intragroup Spearman’s rank correlation coefficient is found to be significant in some groups (PM2.5 vs PM10: (G2: 0.03)]; PM1.0 vs PM2.5: (G2: 0.03)]. The intergroup Spearman’s rank correlation coefficient was found significant for cumulated group TIs among some sites [VLI vs PM2.5 (rs= -0.43; p=0.05); PM2.5 vs PM10 (rs= 0.49; p=0.02); PM1.0 vs PM10 (rs=0.71; p=0.00); PM1.0 vs PM2.5 (rs=0.59; p=0.01). The intergroup Spearman’s rank correlation coefficient for cumulated group TIs among remaining sites was found non-significant for groups namely VLI vs PM10, PM10 vs TVOC, PM10 vs CO_2_ and PM10 vs HCHO (Table 3).

## Discussion

Our study observed range of site mean for G3 (3.93-7.19), G2 (8.05-11.53), G1 (11.69-34.74) are consecutively ascending in nature similar to the observed DT (G3-6.08, G2-9.71, G1-19.03), night time (G3-6.27, G2 -9.60, G1-13.59), and overall group mean (G3-6.17, G2-9.65, G1-16.31) (Table 1). Similarly, a Madurai-based study 17 [9] has specifically underlined the importance of PM10 for vehicle-related pollution. The present study has also found highly increased PM10 in some of the TIs of G1, G2, and G3 groups [(G1/1: PM10, 1369.25); (G2/5: PM10, 113.25), and (G3/2: PM10, 600.65); Table 6]. While this study assessed VLI also as an indicator of traffic intersection-wise pollution (Tables 1, 2, and 7). Similarly, another study [10] conducted during February-March 2012 at Bhopal assessed PM10 and PM2.5 beyond permissible limits but SO_2_ and NO_2_ were within prescribed limits (AQI 105.54, February; AQI 105.89, March). However, the VLI was not assessed therein. The present study has been conducted at different locations of the city. However, the seasonal variation, large scales gatherings, and the annual increase in vehicular pollution due to the use of old vehicles may be limitations of the study.

The range of mean difference for PM10 was 7.2-12.7 μg/m^3^, whereas the mean difference for PM2.5 was 7.9 μg/m^3^ in a study conducted by the Department of Air Quality at The Netherlands [11] indicating 1.3 times higher concentration than the background levels. This study observed group mean for PM10 in descending order among G1, G2, G3 (PM10: (G1: 319.87; G2: 138.00; G3: 51.37) and DT:NT for PM10 for the same order is noted as 2.50, 0.09, and 0.53 (Table 2). It hence shows that, despite the rainy season during the present study, the indicators of air pollution in the city of Bhopal are high as evidenced by high TVOC, CO_2_, HCHO, and PM10 levels of cumulative datasets (Table 6), DT (Table 4), and NT (Table 5) for gaseous particles, particulate matter, and submicron particles assessed during June-September, 2019.

PM10 concentration was found to be raised due to local vehicular traffic in Finland in a study conducted by the Finnish Meteorological Institute [12], whereas PM2.5 concentration was assessed to be high at highways-based transport regions. The present study similarly infers that the increasing traffic load across traffic intersections in the city with expanding developmental initiatives under Capital Development Projects and other private developers shall pose further threats of higher air pollution levels in the years to come with seasonal variations challenging the public health scenario.

Seasonal trends of summer, autumn, winter were assessed in the Republic of Korea [13] by Korea Railroad Research Institute and found significantly high values of pollution indices viz. PM10 (42.5-108.4 μg/m^3^), PM2.5 (61.1-64.0 μg/m^3^), PM2.5/PM10(0.60), PM1.0(50.9- 52.2 μg/m^3^), PM1/PM2.5 (0.79-0.85), and CO_2_ (686.9-701.5 ppm). These findings are in resonance with the findings of the present study. A study in Seoul Metropolitan Subway Stations at Han Yang University, Seoul [14] similarly found PM10 and PM2.5 to be higher than permissible levels of 150 μg/m^3^ and 35 μg/m^3^, respectively, which were significantly higher than those at ground level (p<0.05). These Korean studies highlight the need to generate evidence of air pollution by on-road vehicles and related adverse consequences on human health across the spectrum of demographic profiles. The University of Porto, Portugal [9] collected data of DT and NT for PM10, PM2.5, and PM1.0 and found these to be on a higher level with PM10 (DT mean 125+73 μg/m^3^; NT mean 110+71 μg/m^3^), PM2.5 (DT mean 115+68 μg/m^3^; NT mean 108+70 μg/m^3^), and PM1.0 (DT mean 114+68 μg/m^3^; NT mean 107+69 μg/m^3^). Seasonal trends of summer, autumn, winter were assessed in the Republic of Korea [7] by Korea Railroad Research Institute and found significantly high values of pollution indices, viz., PM10 (42.5-108.4 μg/m^3^), PM2.5 (61.1-64.0 μg/m^3^), PM2.5/PM10 (0.60), PM1.0 (50.9- 52.2 μg/m^3^), PM1/PM2.5 (0.79-0.85), and CO_2_ (686.9-701.5 ppm). These findings are in resonance with the findings of the present study. These Korean studies highlight the need to generate evidence of air pollution by on-road vehicles and related adverse consequences on human health across the spectrum of demographic profiles. The University of Porto, Portugal [15] collected data of DT and NT for PM10, PM2.5, and PM1.0 and found these to be on a higher level with PM10 (DT mean 125+73 μg/m^3^; NT mean 110+71 μg/m^3^), PM2.5 (DT mean 115+68 μg/m^3^; NT mean 108+70 μg/m^3^), and PM1.0 (DT mean 114+68 μg/m^3^; NT mean 107+69 μg/m^3^).

A study conducted at Central Road Research Institute, New Delhi [16] identified PM, VOC, and gas chemicals to be hazardous air pollutants in indoor and outdoor sources. As per sampling done by Grimm Dust Monitor and VOC Monitor at sampling time between 9:30 am and 5:00 pm while noting even the corridor air pollutants level to be alarming in office buildings (PM10: 83.4+44.7 μg/m^3^, PM2.5 65.0+37.3 μg/m^3^, PM1.0 57.8+29.9 μg/m^3^) and VOCs (64.4+21.6 ppm). PM10 concentrations at schools located in city center, residential, and rural area with three classrooms in each (total nine measurement sites) observed PM10 during occupancy to be as (a) school 1: [site 1: (PM10 81.0+11.7 μg/m^3^); site 2: PM10 104+63.5 μg/m^3^); site 3: PM10 70.1+25.2 μg/m^3^)]; (b) school 2: [site 1: (PM10 97.0+15.9 μg/m^3^); site 2: PM10 362+83.7 μg/m^3^); site 3: PM10 177.1+75.0 μg/m^3^)]; (c) school 3: [site 1: (PM10 68.0+19.6 μg/m^3^); site 2: PM10 73.7+13.8 μg/m^3^); site 3: PM10 62.2+8.93 μg/m^3^)] were observed in a study [17]. A study conducted at Indian Institute of Technology, Kharagpur in association with West Bengal Pollution Control Board [18] assessed PM10 and PM2.5 at three sites in Kolkata and observed similar findings [PM2.5 site 1: (96.31-355.19 μg/m^3^); site 2: (116.29-363.63 μg/m^3^); site 3: (99.14-263.0 μg/m^3^) PM10 site 1: (140.5-471.7 μg/m^3^); site 2: (216.21-637.7 μg/m^3^); site 3: (185.42-487.07 μg/m^3^)].

A study of PM1.0, PM2.5, and PM10 was conducted by Clean Air Commission of Vienna, Austria [19] at three urban [site 1: (PM1.0: 14.9+7.7; PM2.5 18.6+10.7; PM10 26.5+13.3); site 2: (PM1.0: 14.7 +8.5; PM2.5 18.8 +12.0; PM10 29.9+19.0; site 3: (PM1.0: 17.5+10.2; PM2.5 21.1+12.9; PM10 31.0+17.0], and one rural site 1: (PM1.0: 12.4+6.1; PM2.5 15.0+8.6; PM10 21.1+10.5) with observation of no seasonal influence at rural site. The present study focused on urban sites during one season only but higher levels of pollution were seen in traffic intersections under the classified categories (Tables 4 and 5). Contrastingly, the study of two Beijing sites, namely Chegongzhuang and Tsinghua, were assessed [20] for PM2.5 and found ranging between 37 and 357 μg/m^3^ and found PM2.5 to be highest in winter and lowest in summer. It is observed in an Australian study [21] that the concentration of particulate matter proportionately decreases for PM2.5 with increasing distance from road reaching to 40% of that level at 150 m distance.

A review report by Central Pollution Control Board, CPCB [22] has noted an average annual exposure level; of PM2.5 to be 34.39, 43.44, and 47 in the years 2000, 2005, 2010, 2011, and 2013, respectively. The present study has noticed PM2.5 vs PM10 (G2: p=0.03), PM1.0 vs PM2.5 (G2: p=0.03), and VLIs PM2.5 (G1+G2+G3: p=0.05).

In the present study, the ratio of DT and NT value assessments for VLI, TVOC, CO_2_, HCHO, and PM10 shows higher proportion in DT for select values as per inter group variability [VLI (G1: 1.4; G2: 1.01); TVOC (G1:2.45; G3:3.66); CO_2 _(G1: 1.3; G2:1.19; G3:1.18); HCHO (G1: 2.69; G3: 03.00); PM10 (G1: 2.5); Table 2].

A study conducted in Italy [23] observed PM10 and PM2.5 at six sites of a town to be in the range of 41.5-89.5 μg/m^3^ and 34.0-62.5 μg/m^3^ from the Advanced Research Project Agencies (ARPA) database. The present study results (Table 3) similarly show the same as Spearman’s correlation coefficient for VLI vs PM2.5 (p=0.05), PM2.5 vs PM10 (p=0.02), PM1.O vs PM10 (p=0.00), and PM1.0 vs PM2.5 (p=0.01) like the seasonal trends of particulate matter from heterogeneous traffic near urban roads assessed at IIT Madras [24] to be higher especially for PM10 concentration as per stated norms of World Health Organization (50 μg/m^3^) and Indian National Ambient Air Quality Standard (NAAQS; 100 μg/m^3^; 2010). The Spearman correlation coefficient (PM10 vs PM2.5; 0.75; PM2.5 vs PM1.0; 0.92, PM2.5-10 vs PM1.0:0.11) was also assessed in a study by National Public Health Institute, Finland [25]. PM2.5 was also found raised as compared to the World Health Organization (25 μg/m^3^) and Indian NAAQS (60 μg/m^3^) during three fourth of the time. The post-monsoon season (PM10:189, PM2.5:84, PM1.0:66 μg/m^3^, winter season (PM10:1135, PM2.5:73, PM1.59 μg/m^3^, summer season (PM10:102, PM2.5:50, PM1.34 μg/m^3^). A Netherland-based study [26] concluded black smoke and NO_2_ concentration to higher near motorways. A study conducted at the University of Dhaka [3] observed mean PM1.0, PM2.5, and PM10 concentration to be 46.1+13.4, 76.0+16.2, 203.9+44.8 μg/m^3^, whereas NO_2_ and TVOC were 0.076+0.007 ppm and 90.0+46.0 ppm in even indoor environment.

A sampling at six locations was conducted for Air Quality Indexing in Bangalore city [27] with AQI ranging 42.64-140.52 (unhealthy for the sensitive group), while noting the parameters like temperature, relative humidity, wind speed, and rainfall. In the present study, the ratio of DT and NT value assessments for VLI, TVOC, CO_2_, HCHO, and PM10 shows a higher proportion in DT for select values as per intergroup variability [VLI (G1: 1.4; G2: 1.01); TVOC (G1: 2.45; G3: 3.66); CO_2 _(G1: 1.3; G2: 1.19; G3: 1.18); HCHO (G1: 2.69; G3: 03.00); PM10 (G1: 2.5); Table 2].

Another study conducted by Southern California Particle Center and Supersite of Centre for Occupational and Environmental Health, Los Angeles [28] measured temperature (Celsius) [summer: 30.3+3.7; winter 23.2+4.0], relative humidity (%) [summer: 66.4+14.8; winter 43.1+21.4], wind speed (m/s) [summer: 1.36+0.66; winter 1.27+0.67], traffic density at 405 freeway (vehicles/min) [summer:231 +30; winter 236+27], traffic density at 710 freeway (vehicles/min) [summer: 203+12; winter 200+11]. The environmental conditions in the present study noted temperature, dew point, humidity wind pressure, wind speed, UV radiation and visibility also with predominantly high humidity (DT: 81.53%, NT: 86.30%), and low visibility (DT: 5.5 unit; NT: 4.85 units; Table 8).

**Table 8:** Mean of various environmental parameters during DT and NT at included TIs under the study (n=20) DT: daytime, NT: nighttime, TI: traffic intersections.

Particulars	Temperature (Celsius)	Dew point	Humidity (%)	Wind pressure (Mb)	Wind speed (Km/H)	UV	Visibility (units)
DT	26.75	24.35	81.35	1003	19.7	Low	5.50
NT	25	24.2	86.3	1004	14.2	Low	4.85

## Conclusions

The present study developed a new index namely VLI, which shall be more realistic to be adopted in the future for assessment of vehicular traffic concentration. The assessed increased levels of PM10, TVOC, CO_2_, and HCHO at all TIs under study including high, medium, and low traffic areas indicate moderate to severe public health threats to the resident community, commuters, nearby schools, and other people-centric facilities. These may lead to cough, asthma, bronchitis, stroke, and premature death among the exposed population as per their demographic and epidemiological profile. The presence of submicron particles (>0.25 μm, >0.3 μm, >0.5 μm, >10 μm) in almost all sites of traffic intersections in DT and NT indicates public health threats due to deposition of these particles into alveoli leading to irreversible pulmonary damage. Hence, there is a felt need for comprehensive strategic pollution prevention and control policy-based initiatives for primary prevention-based public health interventions in varied geological settings, especially in developing nations.
